# Cancer Research in the Time of COVID-19: A Colombian Narrative

**DOI:** 10.3389/fpubh.2021.750755

**Published:** 2022-01-04

**Authors:** Raúl Murillo, Ginna Fernández-Deaza, María Zuluaga, Grant Lewison, Diana Usgame-Zubieta, Iván Darío Usgame-Zubieta, María Margarita Manrique

**Affiliations:** ^1^Centro Javeriano de Oncología, Hospital Universitario San Ignacio, Bogotá, Colombia; ^2^Facultad de Medicina, Pontificia Universidad Javeriana, Bogotá, Colombia; ^3^King's College London, Institute of Cancer Policy, Guy's Hospital, London, United Kingdom; ^4^Dirección de Investigación, Pontificia Universidad Javeriana, Bogotá, Colombia; ^5^Oficina de Investigaciones, Hospital Universitario San Ignacio, Bogotá, Colombia

**Keywords:** neoplasms, research priorities, COVID-19 pandemic, cancer publications, Colombia

## Abstract

Cancer research is deficient in Colombia and efforts and resources diverted due to the COVID-19 pandemic could worsen the situation. We explore the impact of the pandemic on cancer research funding, output, and conduct. We sought information at national level and used the experience of an academic reference center to contrast the impact at institutional level. We searched databases and official documents of national governmental institutions, trial registries, hospital registries, and the Web of Science. We interviewed principal investigators (PIs) to retrieve information on the conduct of cancer research. A decline in resource availability and new proposals was observed at the national level with a shift to COVID-19 related research. However, at institutional level there was no decline in the number of cancer research proposals. The predominance of observational studies as opposed to the preponderance of clinical trials and basic science in high-income countries may be related to the lower impact at institutional level. Nevertheless, we found difficulties similar to previous reports for conducting research during the pandemic. PIs reported long recovery times and a great impact on research other than clinical trials, such as observational and qualitative studies. No significant impact on research output was observed. Alternatives to ensure research continuity such as telemedicine and remote data collection have scarcely been implemented given limited access and low technology literacy. In this middle-income setting the situation shows a notable dependency of international collaborations to develop research on COVID-19 and cancer and to overcome challenges for cancer research during the pandemic.

## Introduction

Colombia is an upper-middle income country with deficient investment in cancer research. The Colombian research and development expenditure as a percentage of the Gross Domestic Product (GDP) was 0.24% in 2018 (1), an investment below most countries in the region with similar GDP.

Although the count of scientific papers for different Latin American countries compared with their gross domestic products shows a positive relationship, the association of cancer research as percentage of biomedical research with cancer as percentage of total disability-adjusted life years (DALYS) in the country is deficient (2). Furthermore, in ordinary times, the Colombian scientific productivity and impact factor around cancer are lower than observed for other Latin American countries with a similar income level.

Two reviews of scientific productivity on cancer research in Colombia found between 1,088 and 1,464 papers published in PubMed/Scielo and Scopus up to 2013 and 2015, respectively, with 290 cancer research groups registered in the governmental scientific platform (Group-LAC) (3, 4). The low productivity (3.8–5.0 papers per group) has been oriented mainly to cancer biology, based on observational data, and focused on cervical cancer. A more recent review revealed an increase in cancer research productivity as measured by the number of scientific publications (1,263 papers between 2014 and 2019), but no major change in research focus and methods (2).

Indeed, Colombia has significantly contributed to the body of knowledge for cervical cancer control, mainly in the field of HPV epidemiology and prevention (5). Consequently, given the improvement of socioeconomic conditions and the long-standing programs on cervical cancer screening, the burden of this disease has significantly decreased (6). However, other leading causes of cancer incidence and mortality are under-represented in the spectrum of cancer research as well as the underlying methods and research approaches requested to properly address their associated burden (2).

In this challenging scenario, the occurrence of a pandemic of an unknown and highly threatening disease diverted efforts and resources, placing an extraordinary burden on decision makers, cancer care providers, and oncology patients. The final impact of such a situation depends upon the institutional strength prior to the pandemic, the ability to adapt to the temporary situation, and the speed of returning to pre-pandemic activities. Certainly, the capability to absorb the extraordinary within the ordinary differs greatly between research centers from high and middle-income economies. Therefore, priority and operational changes during the COVID-19 pandemic may have lasting negative effects on the already deficient development of cancer research in the country.

The first case of COVID-19 in Colombia was reported on March 6th 2020 (7), and a strict mandatory lockdown was instituted from March 24th to August 31st 2020. In this article we aimed to explore the influence of the pandemic on cancer research in Colombia, a middle-income country, using national data and the experience of a reference academic center (San Ignacio University Hospital – HUSI in the Spanish acronym), as related to the restrictive measures and abrupt changes that occurred in the time of COVID-19.

## Methods

We conducted a search in publically available databases and official documents of the Colombian Ministry of Science and Technology (Minciencias) to retrieve information on research applications and approved funding for research in the country during the period January 2019 to June 2021. A manual classification based on title and abstract was done to identify research calls on health subjects. In addition, the objective for all calls including COVID-19 in the title were reviewed to classify calls oriented to health outcomes vs. calls oriented to economic outcomes. Since the available information from Minciencias does not provide details on health topics (i.e., cancer), we carried out an additional search in the databases of ClinicalTrials.gov (condition cancer, country Colombia, all studies) and the Colombian National Institute for Food and Drug Vigilance (INVIMA in its Spanish acronym), the latter including an obligatory registry for studies on pharmacological interventions to be conducted in the country. Since no search terms are available we did a manual search to identify all studies on cancer patients.

For institutional information at the Centro Javeriano de Oncología – Hospital Universitario San Ignacio (CJO-HUSI), we conducted a search on new research proposals and funding in the open source information system Semicrol-Fundanet by using keywords for cancer subjects, medical specialty, and hospital service. We used the terms cancer, carcinoma, tumor, malignant tumor, leukemia, lymphoma, multiple myeloma, hematology malignancies, Centro Javeriano de Oncología, oncology, and radiotherapy. The CJO-HUSI receives about 3,000 new cancer cases per year and is located in Bogota. The HUSI is the academic center of the medical school at the Pontifical Xaveriana University, which has been inexistence for 75 years.

The search on applications and approved projects was supplemented with a bibliometric search of publications in the Web of Science. We used a filter with the titles of 185 specialized cancer journals and 323 title words or phrases related to cancer research for the period January 2019 to June 2021 (2). We also retrieved data for selected domains in cancer research. We collected data on papers with an address in at least one Latin American and Caribbean country to compare with papers with at least one address in Colombia and papers with declared affiliation to the HUSI.

Finally, since there is no theoretical background on the subject we conducted non-structured interviews to explore the experience of selected investigators at CJO-HUSI regarding ethical and administrative approvals, patient recruitment, and data collection. Non-structured interviews gave us the opportunity to ask for specific topics while leaving the possibility for the investigators to provide additional information about an experience without precedents. We collected the information from open questions, interviews were recorded and transcribed. One member of the research team carried out a manual classification regarding impact of workload and task shifting, development and approval of research proposals, difficulties in operational issues, and patient adherence and follow-up. A second member of the team reviewed the initial classification and discrepancies were solved by consensus. Since the purpose of the interviews was limited to gather descriptive information we did not carry out any special coding or qualitative analysis. For the interviews we used a convenient sampling scheme looking for representation of different types of cancer research including surveys and other observational studies, intervention studies, data mining, and qualitative research. We aimed to include at least one PI per research category.

Given the exploratory nature of the analysis all data are descriptive in nature including those collected by using qualitative methods (interviews); thus, we used absolute and relative frequencies to present the results.

## Results

### Research Priorities: The National and Institutional Contexts

**Figure 1:**
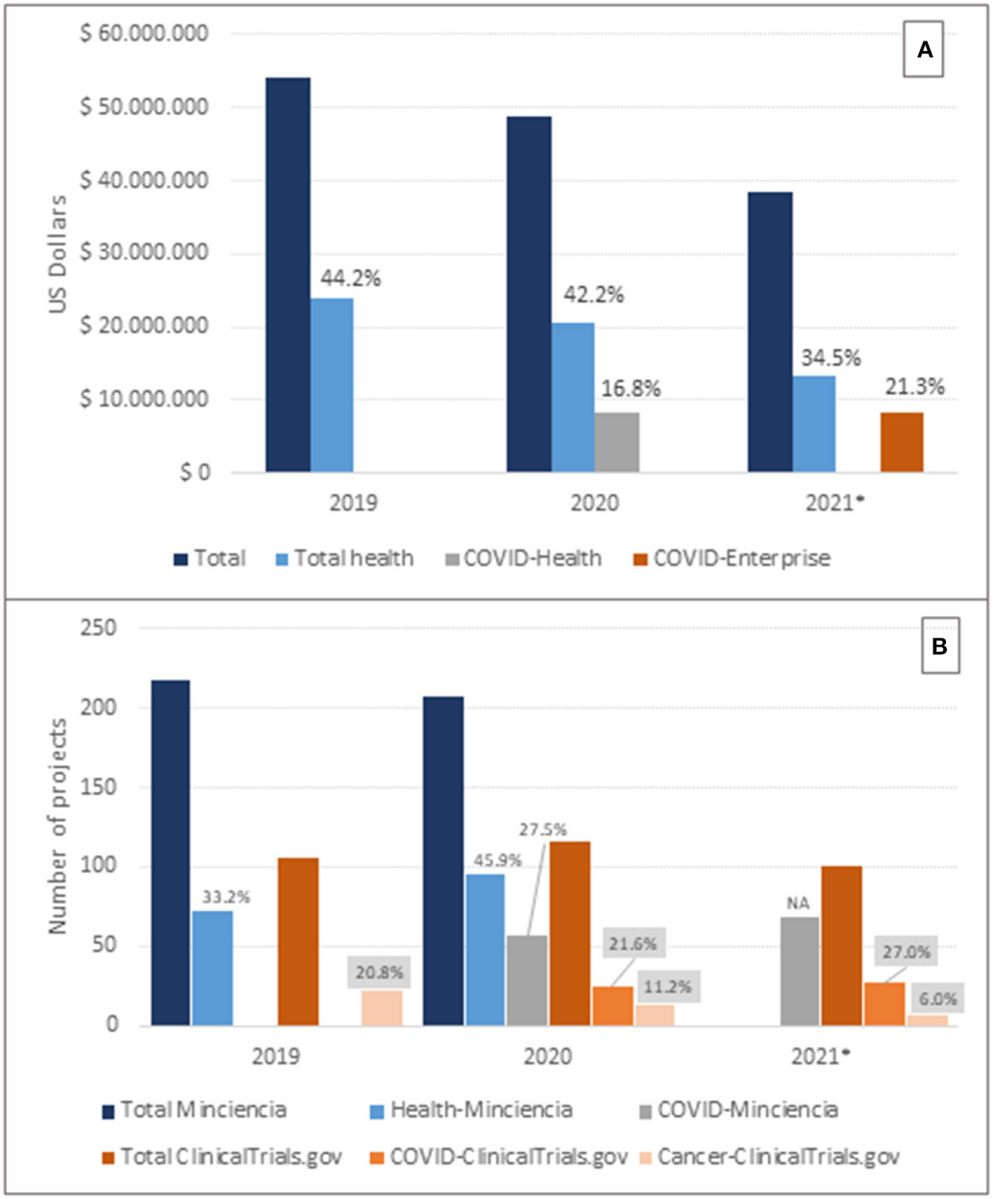
Allocated resources and research projects at the national level. **(A)**: Allocated budget for research and innovation. Source: Minciencias. Percentages referred to the total budget. COVID-Health corresponds to COVID-19 health related research. COVID-Economy corresponds to research and innovation related to the economic recovery during the COVID-19 pandemic. **(B)**: Approved/Registered research projects by data source. Source: Minciencias (Projects funded) (8) and ClinicalTrials.gov (Registered projects by starting date) (9). Percentages should be read with reference to the corresponding total numbers by source. Gray labels refer to numbers in ClinicalTrials.gov. NA: no data available about the total number of projects. *For 2021 partial data up to June 15^th^.

Besides the pharmaceutical industry, the main source of research funds in the country is Minciencias (8). In relative terms, Minciencias increased the budget for research and innovation (177 billion and 180 billion in Colombian pesos for 2019 and 2020, respectively). However, because of the variation in the exchange rate, the total budget decreased by about 5 million for 2020 when estimated in US Dollars (Figure 1A). Furthermore, the participation of the health sector in the total research budget has been reduced (44.2, 42.2, and 34.5% in 2019, 2020, and 2021, respectively), with a significant concentration of resources for research on the COVID-19 pandemic in 2020 and 2021 (Figure 1A).

On the other hand, the participation of the health sector in the total number of projects funded by Minciencias increased in 2020 (Figure 1B), and the total number of studies registered for the country in the database of ClinicalTrials.gov also increased (9). However, according to the latter the participation of cancer research decreased from 20.8 in 2019 to 6.0% in 2021, whereas research about COVID-19 increased from 21.9 in 2020 to 25.0% in 2021 (Figure 1B). Data on COVID-19 research should be interpreted with caution since the reported number of projects by Minciencias in 2021 essentially corresponds to projects within the calls for the economic recovery of the country (Figure 1A), and INVIMA reports only 33 COVID-19 related studies, which are 17 less than the number registered in ClinicalTrials.gov.

At the institutional level, the research fund at HUSI allocated US$394.000 in 2019. However, no resources have been allocated for the years 2020–2021 due to the pandemic. Consequently, no institutional research calls have been launched and the research fund has operated with remnants of the previous year, leading to a reduction of resource availability of about 7.3 in 2020 and 41.7% in 2021, compared with 2019. The available resources have been used to support research training, operation of disease registries, research information systems, intellectual property, and scientific publications.

**Figure 2:**
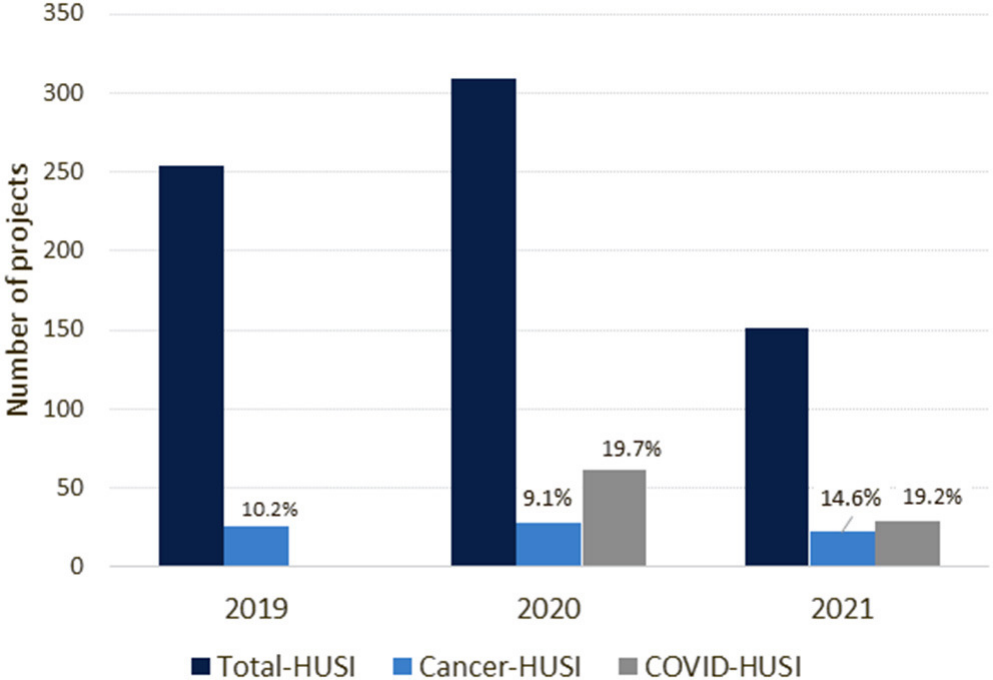
Research proposals registered at HUSI. Source: Semicrol-Fundanet. Percentages should be read with reference to the total number of proposals. *For 2021 partial data up to June 15^th^.

The total number of research projects at HUSI has progressively increased. In total 714 research proposals were registered at the HUSI from January 2019 to June 2021, with cancer related research representing 10.6%. No substantial changes were observed for the number of proposals on cancer research by yearly period; on the contrary, according to partial numbers for the year 2021, an increased participation of cancer research proposals occurred despite the significant amount of COVID-19 related research (Figure 2). During 2020 there were no proposals on both cancer research and COVID-19, but up to June 15th, 2021, two out of 22 projects were registered with concurrent objectives on the two subjects.

Cancer research projects sponsored by the pharmaceutical industry at HUSI numbered two in 2019 and three in 2020, and the same numbers were observed for interinstitutional collaborative research on cancer. For 2021, the request for feasibility analyses for industry-sponsored projects has increased compared with the same period in 2019 (11 and 7, respectively).

### Research Output: Scientific Production

We found no decrease in the global scientific production related to cancer in the Web of Science (Table 1) and no major changes of the participation of the region (Latin America and the Caribbean) or the country (Colombia) in the corresponding immediate context regarding total cancer research. The participation of CJO-HUSI in the Colombian research output on cancer increased from 4.5 in 2019 to 7.8% in 2021, with a similar trend in the research output for palliative care but in this case not restricted to cancer research. On the other hand, there was no scientific production on chemotherapy for the CJO-HUSI in 2021; this corresponds to the decreasing trend for the participation of Colombia in the regional context for this specific domain.

**Table 1:** Scientific production in cancer.

**Domain**	**Number of scientific papers**			**Participation (%) in the immediate context**		
**Cancer (all-inclusive)**	**2019**	**2020**	2021^*^	**2019**	**2020**	2021^*^
World	226,335	235,920	106,908	ref	ref	ref
Latin America and Caribbean	6,688	7,388	3,204	3.0	3.1	3.0
Colombia	529	542	218	7.9	7.3	6.8
CJO-HUSI	24	30	17	4.5	5.5	7.8
**Chemotherapy**						
World	48,981	50,397	22,549	ref	ref	ref
Latin America and Caribbean	2,030	2,095	984	4.1	4.2	4.4
Colombia	126	112	49	6.2	5.3	5.0
CJO-HUSI	1	4	0	0.8	3.6	0.0
**Radiotherapy**						
World	25,594	25,611	11,680	ref	ref	ref
Latin America and Caribbean	761	870	391	3.0	3.4	3.3
Colombia	40	55	23	5.3	6.3	5.9
CJO-HUSI	0	2	1	0.0	3.6	4.3
Palliative care^**^						
World	71,568	77,719	37,727	ref	ref	ref
Latin America and Caribbean	3,389	3,763	1,727	4.7	4.8	4.6
Colombia	288	343	140	8.5	9.1	8.1
CJO-HUSI	1	11	6	0.3	3.2	4.3

*Participation in the immediate context corresponds to percentage estimates as related to total numbers in the corresponding level: Latin America compared with the World, Colombia compared with Latin America, and CJO-HUSI compared with Colombia*.

** *Includes all diseases, not only cancer*.

* *For 2021 partial data up to June 15^th^*.

### Cancer Research Approach at Institutional Level

We observed no major changes in the direction of cancer research at HUSI with the exception of a decrease in basic science (23.1, 10.7, and 4.5% in 2019, 2020, and 2021, respectively). Observational studies are the most prevalent (67.9 in 2019, 75.9 in 2020, and 90.9% in 2021), and most of them corresponded to descriptive studies (50% of cancer research for both 2019 and 2020, and 45.5% up to June 2021); however, analytical studies increased in 2020 and more markedly in 2021. Intervention studies represented 10.1, 7.3, and 4.5% for the years 2019, 2020, and 2021 (Supplementary Figure).

**Figure 3:**
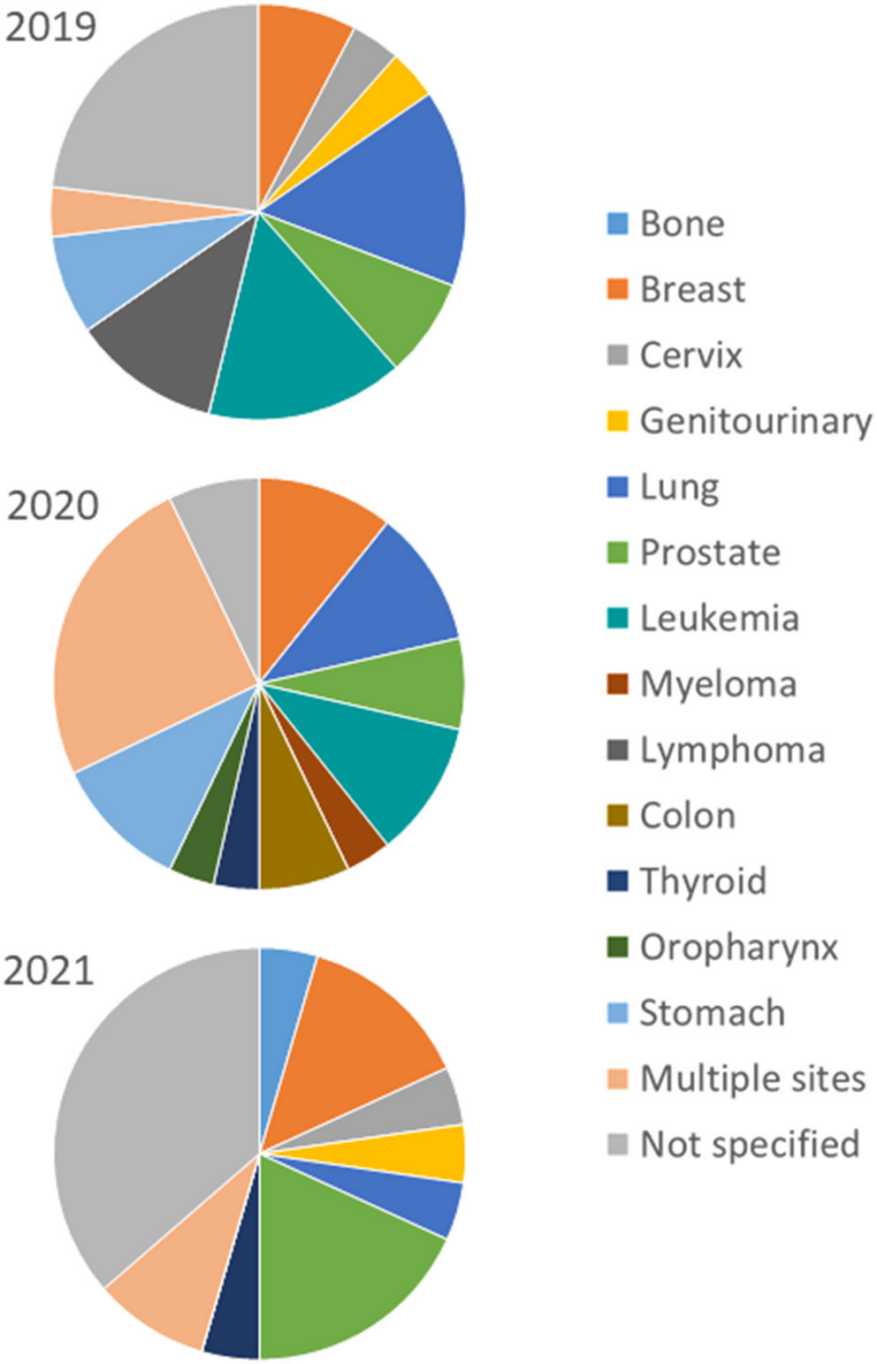
Cancer research by anatomical site at HUSI. Source: Semicrol-Fundanet. *For 2021 partial data up to June 15^th^.

For the period analyzed most projects on cancer research were not oriented to specific anatomical sites (only 21.1%). Furthermore, the second most frequent category corresponds to multiple anatomical sites (13.2%) clustered mainly on gynecological, gastrointestinal, hematological and eye-and-orbit neoplasia. For specific anatomical sites, breast, lung and prostate cancer were the most researched in equal proportions (10.5%), followed by leukemia (9.2%), and stomach cancer (6.6%). There was a progressive decline in participation from 2019 to 2021 in lung cancer (from 15.4 to 4.5%), lymphoma (from 11.5% to nil), and leukemia (from 15.4% to nil). By contrast, prostate cancer research increased markedly in 2021 (from 7.7 up to 18, 2%) (Figure 3).

### Operational Impact on Cancer Research

#### Approval and Ethical Regulation

In order to reduce the time for evaluation and approval, the Ministry of Health and INVIMA allowed the conduct of COVID-19 related trials in research centers without certification in Good Clinical Practice (GCP) but under the guidance of certified centers (10). Furthermore, INVIMA made recommendations on remote research activities including remote electronic informed consent, domicile biospecimen collection and drug delivery, remote monitoring, and biosecurity measures for participants and research teams (11).

Moreover, from March 16th to July 31st 2020, the IRB at HUSI indicated the mandatory deferral of active recruitment and the suspension of procedures for research projects with non-COVID objectives, if requiring the participants to travel during the confinement to the research center exclusively for research purposes, exception made of necessary activities to ensure patient safety and treatment delivery.

In addition, the IRB at HUSI indicated the mandatory introduction of a new document to inform participants about risks and rights regarding their participation in research during the COVID-19 pandemic. As a consequence of the field work deferral, the institutional research monitoring system for safety and data quality had reduced interaction with research teams during 2020, with a decreased number of monitoring visits (40% less than in 2019). This also related to the augmented duties for regular clinical activities during the COVID-19 pandemic, which engendered a reduced availability for research activities. As a response to the extraordinary conditions, remote monitoring was strengthened by implementing virtual environments and optimizing the use of remote electronic health recording.

For intervention studies, the Research Office at HUSI defined a contingency plan for ongoing projects to ensure treatment continuity, as well as safety and follow-up. The strategies included remote care (telemedicine, home visits) and transport to the research center. After the deferral declared by the IRB remote informed consent has been implemented by nine studies.

#### Patient Recruitment

Principal investigators report a significant delay in participant recruitment and biospecimen collection due to both the strict confinement and the deferral of field work for already-approved research. After resuming field work activities, a reduced recruitment pace has been observed mainly related to lower attendance to health centers, reduced willingness to participate in research, and more time needed to complete the recruitment process.

Besides the deferral as indicated by the IRB, certain sponsors providing external funding initiated a temporary suspension of recruitment not necessarily aligned with the deferral request at HUSI, thus causing additional delay not only in patient recruitment but also in treatment delivery.

Additional factors associated with reduced recruitment rates for ongoing research were the change in patients' eligibility due to modification of standard clinical protocols aimed at reducing the risk for cancer patients during the pandemic, and the delay in international shipment of biospecimens for collaborative studies which restrained recruitment in order to ensure the viability of sample analyses.

#### Data Collection

Variation in methods for data collection led to prolonged schedules, reallocation of resources to new requirements such as phone calls and educational material, and additional tasks to adjust instruments and retrain research teams to the new methods.

Changes such as replacing in-person surveys or interviews by phone calls or web-based questionnaires reduced response rates from about 80 to 30% and represented greater challenges to ensure participants' confidence to provide personal data and detailed information about the disease and the treatment when needed.

Regarding qualitative data collection, face-to-face in-depth interviews have been frequently declined and appointments for virtual interviews frequently missed. Moreover, collection of additional information such as that derived from non-verbal language and the expected feedback from participants to researchers have been more difficult. The arrangement of in-person focus groups has not been possible and the proposed change to virtual focus groups has not been successful despite a reduction in the expected number of participants.

Finally, despite the availability of databases for studies based on data mining, given the deferral declared by the IRB for already approved research and the temporal suspension of acceptance of new proposals, in some cases access to these data was delayed with the corresponding impact on project schedules.

## Discussion

We observed a reduced availability of resources for health research and a reduced participation of cancer research projects in the national context with a corresponding shift to COVID-19 related research. Similarly, the allocated budget for research significantly decreased in the reference center analyzed (HUSI), but in this case, cancer research projects preserved their participation in the global spectrum of institutional research. The lack of impact at institutional level as opposed to the situation in the national context might be related to the nature of cancer research. Most cancer research at HUSI corresponds to observational studies which may require lower budgets and are less restrictive in ethical terms than intervention studies or basic science projects.

Although the limited impact on new cancer research proposals, the interruption of the field-work in ongoing research posed significant challenges for patient recruitment and follow-up as well as for protocol and GCP compliance and adherence (12, 13). Despite new ways of approaching research during the pandemic such as the use of telemedicine and remote methods for data collection (14), the limited access and technology literacy in Colombia represent a challenge for this purpose given that broadband connectivity is 61.0 with only 37.5% of Colombian homes having a permanent subscription to an internet provider (15), and real access based on the availability of proper hardware could be as low as 16.7% in certain regions and socioeconomic contexts in the country (15). Accordingly, only a few studies at HUSI have taken the opportunity to use remote data collection and patient recruitment.

Furthermore, remote research activities may pose greater challenges for certain types of study. The needs for informed consent, treatment delivery, outcomes measurement and data collection have been carefully analyzed for the conduct of clinical trials remotely (14, 16). However, the need for closer interaction in qualitative research and the reluctance to participate in remote qualitative methods of data collection represent a threat to the integrity of this type of research.

The operational difficulties found are similar to those in previous reports about the impact of the COVID-19 pandemic on cancer research (17, 18), but the capacity to overcome such challenges and the limitations of the context made recovery slower. It would be different from that in high-income countries where basic science and clinical trials have a greater presence in cancer research, and consequently, a greater impact of priority changes due to the COVID-19 pandemic (19–21). Indeed, some trial registries observed a reduction down to 60% in launches after the beginning of the pandemic (21). While reactivation of intervention studies in oncology occurred speedily in high-income countries (16, 22), the interviewed investigators in our analysis reported the effect of these challenges for recruitment and follow-up remaining after resuming research activities.

Moreover, in our analysis the institutional measures to protect health research participants had an impact on all types of research regardless of their nature or associated risk. The reduced recruitment rates and the delay in research activities compromise not only the integrity of institutional research but also its compliance with objectives and schedules in national and international collaborations as well as the sustainability of research projects. This situation might have lasting negative effects for future collaborations and research capacity strengthening if not properly addressed.

For the moment, we found no decrease of scientific productivity of cancer research in either context but a slight change in the focus as indicated by the analysis in selected domains (Table 1 and Figure 3). These findings are consistent with detailed analysis of global cancer research during the pandemic which indicates no decline in the research output but a shift in research domains and types of cancer investigated (23). In fact, the institutional participation in the national context increased during the period analyzed, but this trend is not likely to be associated with changes during the pandemic. Furthermore, published research about COVID-19 and cancer indicates that most studies have been conducted in high-income countries (72%) (23).

We observed only a small number of proposals on cancer and COVID-19 at HUSI which may stem from limited institutional capacity for a rapid reaction, thus indicating a dependence on international collaboration. This situation differs from the significant investment in and participation of COVID-19 related research at national and institutional levels. Unfortunately, we did not find specific information on research about COVID-19 and cancer at the national level, but in the institutional database we found only one collaborative project led by the Colombian Association of Hematology and Oncology (ACHO), and given the existence of a close relationship between CJO-HUSI and ACHO with participation in several cancer research projects led by the latter, the finding suggests limited research activity on cancer and COVID-19 in the national context as well.

Within this complex scenario international collaborations may help adopt some of the recommendations already in the literature (24, 25); in addition, strengthening alliances with national stakeholders including health insurance companies, cancer centers, scientific societies, and academic institutions, would increase the feasibility of a more rapid response in research and innovation to threatening conditions such as the COVID-19 pandemic. Furthermore, already existing liaisons between academic centers like HUSI and economic institutions like the Chamber of Commerce or the Inter American Bank, represent an opportunity to enhance resource availability and properly address research questions with connection with the two major areas of interest during the pandemic, namely health and the economy, embracing cancer-related research.

Our analysis has several limitations. The inclusion of only one academic center restrain external validity. Although CJO-HUSI is not representative of all cancer centers, it was the leading health institution regarding research and innovation in Colombia during 2020 according to the Scimago ranking (26) and we think it is likely to parallel the situation in many centers from similar settings. Our exploratory analysis might be reduced in terms of participants in the interviews; however, we consider we properly captured challenges and limitations of conducting cancer research in the restrictive context induced by the pandemic. Unfortunately, we found no detailed real life information from low and middle income countries since almost all publications essentially provide recommendations based on good theoretical backgrounds; in consequence we were not able to contrast our findings with the experience from similar settings beyond data on research output.

## Data Availability Statement

The raw data supporting the conclusions of this article will be made available by the authors, without undue reservation.

## Ethics Statement

Ethical approval for this study and written informed consent were not required in accordance with local legislation and national guidelines. The Colombian regulation for conducting research in human subjects is consigned in the Resolution 8430 de 1993. This study did not involve human subjects since it is an institutional analysis. In addition, the study did not collect or use identifiable human data for any purpose.

## Author Contributions

RM: design, data collection, data analysis, first-draft writing, and writing review and editing. GF-D: data collection, data analysis, and first-draft writing. MZ: data collection and first-draft writing. GL: design and writing review and editing. DU-Z and IU-Z: data collection, data analysis, and writing review and editing. MM: design, data collection, data analysis, and first-draft writing. All authors contributed to the article and approved the submitted version.

## Conflict of Interest

The authors declare that the research was conducted in the absence of any commercial or financial relationships that could be construed as a potential conflict of interest. The handling editor declared a shared committee with one of the authors RM at time of review.

## Publisher's Note

All claims expressed in this article are solely those of the authors and do not necessarily represent those of their affiliated organizations, or those of the publisher, the editors and the reviewers. Any product that may be evaluated in this article, or claim that may be made by its manufacturer, is not guaranteed or endorsed by the publisher.
