# Supplementary figures and images for: Cancer Research in the Time of COVID-19: A Colombian Narrative
Source: Front Public Health. 2022 Jan 4;9:750755. doi: 10.3389/fpubh.2021.750755 (PMC8764311; doi:10.3389/fpubh.2021.750755)

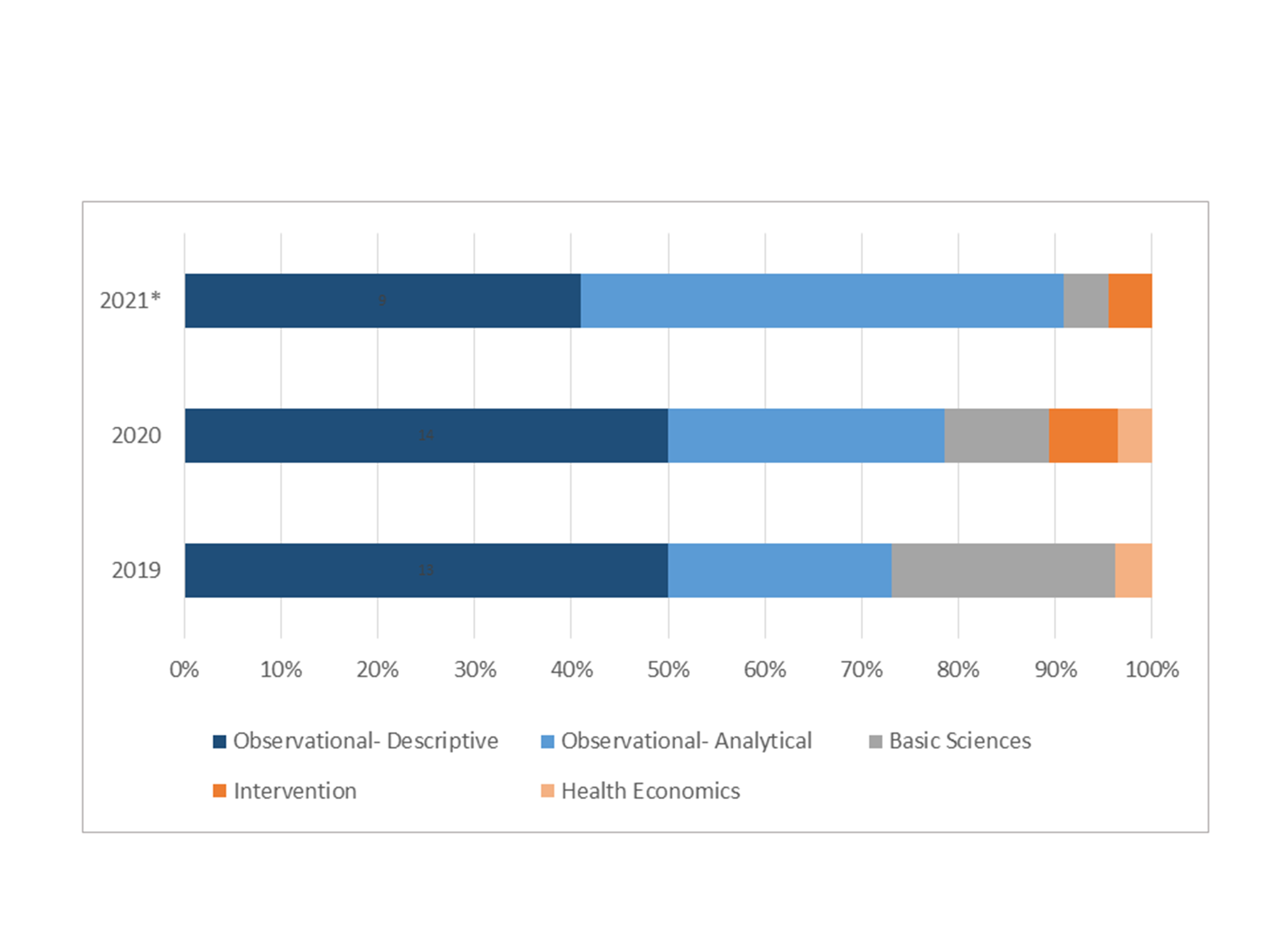

Supplement: Supplementary file 1 [file Image_1.TIF]
